# Bilateral vestibulopathy patients’ perspectives on vestibular implant treatment: a qualitative study

**DOI:** 10.1007/s00415-021-10920-z

**Published:** 2021-12-11

**Authors:** Lisa van Stiphout, Florence Lucieer, Nils Guinand, Angélica Pérez Fornos, Maurice van de Berg, Vincent Van Rompaey, Josine Widdershoven, Herman Kingma, Manuela Joore, Raymond van de Berg

**Affiliations:** 1 Department of Otorhinolaryngology and Head and Neck Surgery, Division of Balance Disorders, School for Mental Health and Neuroscience, Maastricht University Medical Center, Maastricht, The Netherlands; 2 Service of Otorhinolaryngology Head and Neck Surgery, Department of Clinical Neurosciences, Geneva University Hospitals, Geneva, Switzerland; 3 Department of Otorhinolaryngology and Head and Neck Surgery, Faculty of Medicine and Health Sciences, Antwerp University Hospital, University of Antwerp, Antwerp, Belgium; 4 Department of Clinical Epidemiology and Medical Technology Assessment (KEMTA), Maastricht University Medical, Maastricht, The Netherlands; 5 Care and Public Health Research Institute (CAPHRI), Maastricht University, Maastricht, The Netherlands

**Keywords:** Bilateral vestibulopathy, Patient expectations, Vestibular implant, Qualitative study

## Abstract

**Objectives:**

The aim of this study was to explore expectations of patients with bilateral vestibulopathy regarding vestibular implant treatment. This could advance the definition of recommendations for future core outcome sets of vestibular implantation and help to determine on which characteristics of bilateral vestibulopathy future vestibular implant research should focus.

**Methods:**

Semi-structured interviews were conducted with 50 patients diagnosed with bilateral vestibulopathy at Maastricht UMC + . Interviews followed a semi-structured interview guide and were recorded and transcribed. Transcripts were analyzed thematically by two independent researchers. A consensus meeting took place to produce a joint interpretation for greater dimensionality and to confirm key themes.

**Results:**

Overall, patient expectations centralized around three key themes: (physical) symptom reduction, functions and activities, and quality of life. These themes appeared to be interrelated. Patient expectations focused on the activity walking (in a straight line), reducing the symptom oscillopsia and being able to live the life they had before bilateral vestibulopathy developed. In general, patients indicated to be satisfied with small improvements.

**Conclusion:**

This study demonstrated that patient expectations regarding a vestibular implant focus on three key themes: symptom reduction, functions and activities, and quality of life. These themes closely match the functional improvements shown in recent vestibular implantation research. The results of this study provide a clear guideline from the patient perspective on which characteristics of bilateral vestibulopathy, future vestibular implant research should focus.

**Trial registration:**

NL52768.068.15/METC

**Supplementary Information:**

The online version contains supplementary material available at 10.1007/s00415-021-10920-z.

## Introduction

Bilateral vestibulopathy (BV) is a chronic disorder with deficits of the vestibular organs, the vestibular nerves, and/or the brain, which leads to bilaterally reduced or absent vestibular function [1, 2]. Symptoms include unsteadiness when walking or standing, worsening of unsteadiness in darkness or on uneven ground, and movement-induced blurred vision (oscillopsia) [3]. Additional symptoms such as depression, anxiety, cognitive impairment, and an increased risk of falling can also occur [4–8]. All these symptoms might have a strong negative socio-economic impact and can lead to a deterioration of quality of life [7, 9–11].

Currently, evidence for an effective treatment for BV in the clinical practice is lacking [9, 12–14]. However, it has been demonstrated that partial artificial restoration of the vestibular function, in particular the vestibulo-ocular reflex, the vestibulo-collic reflex, and postural responses, is possible for BV patients through electrical stimulation of the vestibular nerve using a vestibular implant [15–18]. It was shown that the vestibular implant is able to provide a functional benefit: the visual acuity in dynamic conditions could be improved and even normalized for patients suffering from BV [19, 20]. These results suggest that a vestibular implant may be able to address the major symptoms of BV patients like oscillopsia and postural control [16, 18]. Therefore, the vestibular implant appears to be a promising treatment strategy for patients with BV [21].

Until now, evaluating the feasibility of restoring controlled vestibular responses has been the main purpose for vestibular implant research. However, little is known about the expectations and preferences of BV patients with respect to receiving a vestibular implant. Previous studies in other research fields showed that patients with high expectations of treatment outcomes were more likely to present better outcomes after treatment, in comparison with patients with low treatment expectations [22–25]. In contrast, other research showed that high expectations can have a negative association with the self-reported benefit of interventions [26, 27]. The existence of contradicting evidence about the correlation between high expectations and treatment outcomes suggests that this correlation might be determined by the investigated condition or disorder.

Regarding the vestibular implant, studying the expectations and preferences of BV patients is an important step in determining the research agenda. By doing so, the patients’ perspective with regard to any type of vestibular implant can be determined. This could advance the process of defining recommendations for future core outcome sets of vestibular implantation and help to determine on which BV characteristic(s) future vestibular implant research should focus. Moreover, it could define the actual clinical value of the vestibular implant for the rehabilitation process. In other words, are patient expectations in line with the potential functional improvements shown in recent vestibular implantation research? Therefore, the objective of this study was to explore and evaluate expectations of BV patients regarding the vestibular implant. It was hypothesized that expectations of vestibular implant treatment outcomes cluster in several key themes, comparable to the themes of BV symptom presentation (physical, emotional, and cognitive symptoms) [7–9]. Next to this, it was hypothesized that the overlapping theme of patient expectations of vestibular implant treatment outcomes would focus on improvement of overall well-being [28].

## Methods

### Patients

Patients with BV diagnosed at the Maastricht University Medical Center + (MUMC +) were asked via mail to participate in this study. Inclusion criteria for BV included imbalance and/or oscillopsia during walking or head movements, and a reduced bithermal caloric response (mean peak slow phase velocity < 6°/s bilaterally) and/or a reduced vestibular-ocular-reflex (VOR) gain as measured by horizontal video Head Impulse Test (bilateral VOR gain < 0.6) and/or torsion swing test (VOR gain < 0.1). A qualitative approach consisting of one-to-one interviews with patients with BV was used to best evaluate patients’ expectations. The setting in which these patients were included and interviewed consisted of a full day of clinical tests in the context of a prospective cohort study for the characterization of BV. Sampling was therefore consecutive (i.e., each consecutive patient diagnosed with BV at MUMC + was approached for enrollment). A potential patient who was not able (e.g., mentally disabled) or willing to talk about one of the investigated issues (e.g., psychology/psychiatry, health care utilization), was not able to stop medication against anxiety or depression (because of its influence on the vestibular test results), or willing to undergo one of the detailed physical, audiometric, or vestibular examinations, was excluded from participation in this study. There were no dropouts.

### Data collection and thematic analysis

The interviews followed a semi-structured interview guide to ensure that the same topics were addressed in all interviews from July 2016 to July 2018 (Online Resource 1). Open questions focused primarily on patient expectations regarding treatment outcome prior to receiving a vestibular implant. To gain additional information about the interpretation and understanding of patients’ statements, the interviewer probed and prompted further into the patients’ responses. After gathering information about patient expectations, the interviewer was free to follow respondent-driven topics and the items discussed could therefore differ between patients. The respondent-driven topics were related to the expected or acceptable number of post-operative care consultations, acceptable or unacceptable surgical risks, the perceived personal value of the vestibular implant, and the desired level of overall improvement after receiving a vestibular implant. All interviews were conducted in Dutch by the same researcher (FL), took place in a hospital setting, and lasted 30–90 min. Interviews were audio-recorded, anonymised, and transcribed verbatim. The transcripts were coded and analyzed thematically by two independent researchers (LvS and MvdB). An inductive approach was used with repeated rounds of reading and categorizing to derive patterns within the data and to identify recurrent themes across the dataset [29]. Both researchers read the transcripts line-by-line and used open coding to manually code data extracts (e.g., words and/or statements) and categorized these thematically. Constant comparison of the categories, codes, and re-reading transcripts ensured the identification of the final themes. Data collection was performed beyond the point of inductive thematic saturation to gain a more in-depth understanding of the emerged themes [30]. A consensus meeting took place (LvS and MvdB) to discuss discrepancies in coding or categorizing to produce a joint interpretation for greater dimensionality. Finally, the summary descriptions of identified themes were formulated in consensus [7, 9]. Neither the interviewer nor the independent researchers conducting the analysis had established patient relationships prior to conducting this study. Illustrative quotes were used to represent the findings. The methods, findings, analysis, and interpretations are reported in accordance with the consolidated criteria for reporting qualitative research (COREQ; Online Resource 2) [31].

## Results

### Patient characteristics

Fifty patients (50% female, mean age 60 years (range 21–79 years)) with BV diagnosed at MUMC + were included in this study. In 58% of the included patients, a definite etiology could be determined, with ototoxicity as the most common etiology (38%). In 22%, the etiology was of probable cause, and in 20%, the etiology was idiopathic. A bilaterally reduced caloric response was found in 90% of the patients (sum of bithermal SPV < 6°/s in each ear), while 78% had a bilaterally reduced VOR gain measured with vHIT (< 0.6), and 60% had a reduced VOR gain on torsion swing test (< 0.1). Fifty-two percent of the patients met three of the vestibular inclusion criteria described above.

### Themes of patients’ expectations

**Fig. 1:**
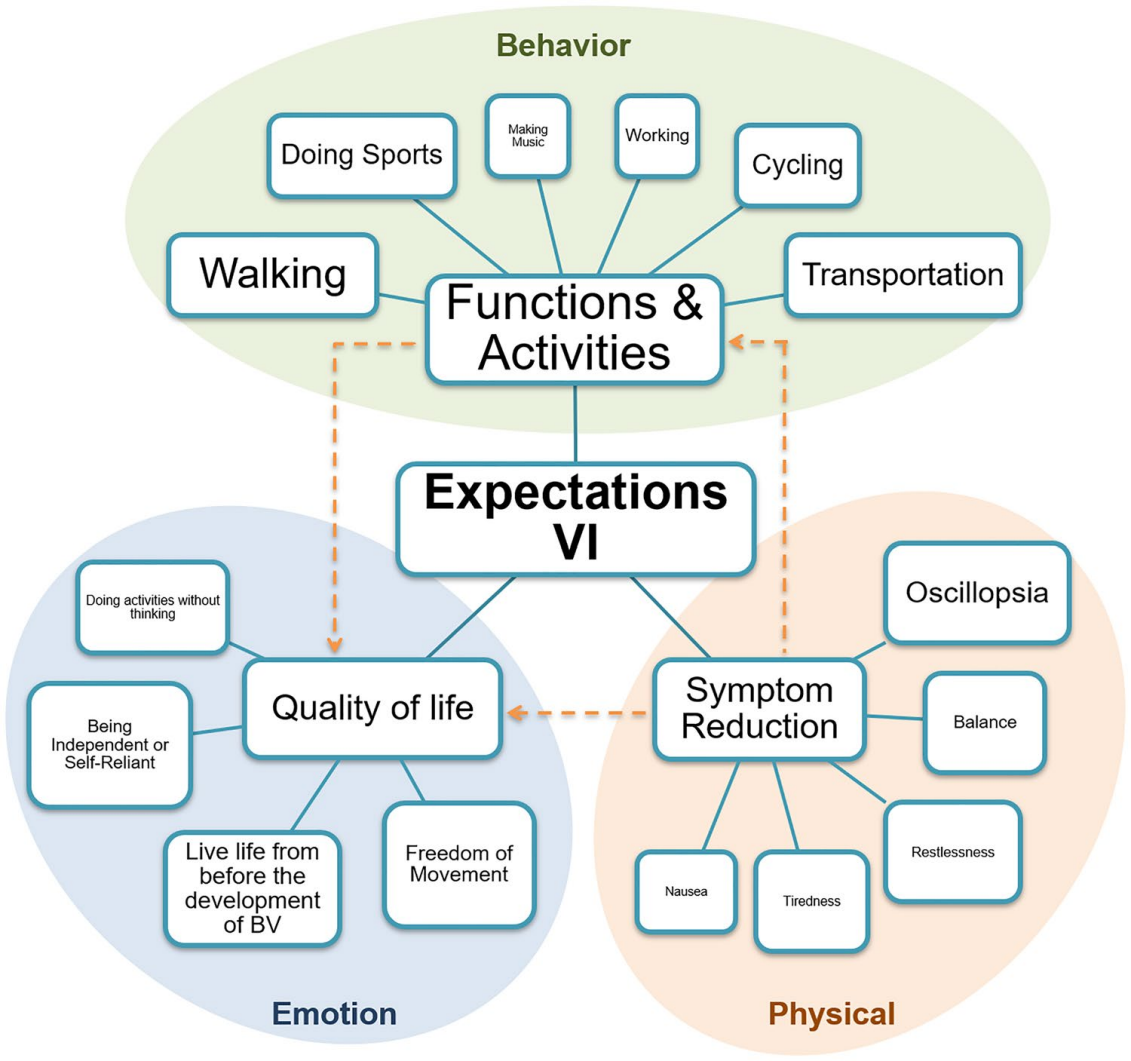
Schematic representation of expectations of patients with BV regarding the vestibular implant (VI) in three key themes: functions and activities, symptom reduction, and quality of life. The larger the font of the text, the more often that expectation was expressed. Arrows indicate the interrelation between themes. Oval planes represent the overarching domains

Analysis of all 50 transcripts revealed diverse, explicit, but also more nuanced expectations about vestibular implantation. Three key themes were identified: symptom reduction, functions and activities, and quality of life (Fig. 1). Each theme was related to an overarching domain: the physical domain (symptom reduction), the behavioral domain (functions and activities), and the emotional domain (quality of life).

*The first theme* clustered around symptom reduction. Patients expressed several expectations focused on symptom relief. Within this domain, reduction of oscillopsia was frequently mentioned:

‘It would be nice to be able to walk and look around at the same time.’ [P20]﻿

‘I think I would be very happy if images would stand still. I find that the most disturbing.’ [P43]

followed by reduction of imbalance:

‘I would like to just get my stability back. I think my problem would almost be solved when I have my stability back.’ [P24]

No explicit expectations regarding the improvement of cognitive or emotional symptoms, such as problems with performing dual tasks or having anxiety, were mentioned. Symptom reduction is, therefore, mainly focused on the physical burden of BV (Fig. 1: Physical domain).

*The second theme* concentrated on gaining function and resuming activities. Patients expressed their expectations by means of resuming one or more activities. To resume walking (in a straight line) was commonly pointed out:

‘To no longer have those swings during walking, that I could just walk in a straight line. That would be nice, yes.’ [P13]

followed by other types of transportation, doing sports and cycling:

‘I would love to drive again. My wife drives really well, but I would prefer to drive myself.’ [P23]

‘We always liked to dance and that is no longer possible. I have lost my balance. So yes, now I just stand next to a table.’ [P28]

‘I would like to be able to cycle. I always liked that. I sat on that stupid thing with my eyes closed!’ [P34]

These activities were often related to social life, previous work, hobbies, or activities of daily living. This second theme, function and activities, was therefore clustered around the overarching domain behavior (Fig. 1):

‘Walking. I have always walked a lot. Twice a year, I went to the Ardennes [region in Belgium and France with extensive forests and rolling hills] with ten friends. We had an apartment there and it was very cosy and very nice. But now I can no longer walk in the woods. Not in the forest at all.’ [P16]

*The third theme* clustered around quality of life. Explicit but also more nuanced expectations focused on improvement of quality of life were expressed. Patients expected an improvement of their quality of life, by being able to live the life from before they developed BV:

‘Everything. I would like to live the life I used to live again. Being busy. Having people around me. I would like that again. But that is not possible now.’ [P53]

followed by gaining more freedom of movement:

‘I would like to regain my freedom in the evening. Get back your freedom of movement. Yes, that would be nice.’ [P12]

and by being more independent or self-reliant:

‘That you are independent of other people. Self-reliant.’ [P19]

Although nobody explicitly indicated an expectation in improving the emotional burden of BV, the expectations within the theme quality of life showed connections with emotions such as insecurity, worry, guilt, or frustration (Fig. 1: Emotional domain):

‘But just the fact that you are thinking about it that way, and then you enter the room and you don't know everyone right away. You need to look where they are. That's just annoying and you just don't want to. You would just prefer to simply walk or cycle over there and also walk in there without worrying that you might go to the left or to the right or you know. I just find that annoying.’ [P20]

A detailed overview of the three key themes and their underlying aspects discussed above can be found in table 1 in Online Resource 3.

Connections were also established between the different themes: patients often discussed how improvements of an aspect underlying one theme would affect aspects underlying another theme. For example, connections were made between balance or oscillopsia (symptom reduction) and resuming activities like walking or cycling. Another illustration was the connection between being able to travel or drive a car again (resuming activities) and therefore being more independent or self-reliant (improvement of overall quality of life):

‘Being able to travel by public transport. Being able to board the train by myself, so that I can go to my daughter independently. Just whenever I want.’ [P19]

### Respondent-driven topics

The analysis showed overlap of respondent-driven topics that were regularly discussed in different interviews. These respondent-driven topics were related to the perceived personal value of the vestibular implant, the desired level of overall improvement after receiving a vestibular implant, the expected or acceptable number of post-operative care consultations, and acceptable or unacceptable surgical risks.

#### Modest expectations versus high value

The desired level of overall improvement after receiving a vestibular implant was discussed by 15 patients, of which some patients indicated that they desired 100% improvement. However, other cases showed more variation in the level of desired improvement, ranging from 70% to very little improvement:

‘Every small improvement would be a bonus.’ [P02]

‘If I look at how I am feeling now, then a little improvement would also be of help. If it improves with 50%, then it would be a lot better. Then you have more quality of life again. Then you can do more things. Especially the images I see, suppose it would be possible that the images I see stabilize a bit better, not entirely, then it is already much better. Say from 50% to only 5%. Yes, then I say; you can learn to live with that, that is manageable. Now it is still too much actually. I think all the small improvements are going to help.’ [P44]

Whether the patient would consider a vestibular implant at the moment of the interview was discussed by 19 patients, of which most patients mentioned they would definitely consider a vestibular implant at that moment. However, some patients would not consider a vestibular implant at the moment of the interview. Some of these patients indicated that they could still manage in daily life and one patient was insecure about potential complications related to the surgery.

Patients also expressed an opinion regarding the personal value of the vestibular implant. Patients stated that a treatment like a vestibular implant would be priceless or would be worth paying a high price for (in proportion to the economic status), with or without the support of health insurance:

‘I would do anything to get better. I cannot put a number on it. But it is worth everything to me.’ [P16]

‘One hundred, two hundred, three hundred thousand euros. Quite a lot for our standard [of living] anyway.’ [P19]

However, some patients explicitly indicated that they would like to have the vestibular implant reimbursed via health insurance and one patient mentioned clearly that a vestibular implant is not worth everything.

A detailed overview of the desired level of overall improvement, the proportion of patients who would consider a vestibular implant, and the estimated personal value discussed above can be found in Table 2, 3 and 4 in Online Resource 3.

#### Acceptable post-operative care

Expectations about the organization or number of post-operative care consultations were mentioned by 18 patients. Patients reported differences in the expected post-operative control consultations, ranging from a weekly to a half-yearly interval:

‘If I would qualify for an implant, I am prepared to do a lot. If I have to stand on the hospital’s doorstep every week, then of course, I will do that.’ [P22]

A detailed overview regarding the number of post-operative care consultations discussed above can be found in Table 5 in Online Resource 3.

#### (Un)acceptable risk factors

Some acceptable risk factors, such as post-operative infections or single-sided deafness, were mentioned during the interviews:

‘It would be a desirable thing to me. If you can get your balance back in exchange for deafness in one ear, then that will be a relief.’ [P35]

However, patients were more explicit about unacceptable risk factors, of which deafness (not specified as uni- or bilateral deafness) was frequently mentioned:

‘But for the implant treatment I have to know for sure that I would not lose my hearing. I don't want to try to solve one thing by damaging another.’ [P17]

Some patients did specify the laterality of hearing loss: they strictly indicated bilateral and/or unilateral deafness as an unacceptable risk factor. Other unacceptable risk factors pointed out were other (unspecified) organ damage, blindness, paralysis, amnesia, or infections. A detailed overview regarding the (un)acceptable risk factors can be found in Table 6 in Online Resource 3.

## Discussion

This study investigated the expectations of patients with BV regarding vestibular implant treatment by performing a qualitative study using semi-structured interviews among 50 patients. Overall, patient expectations centralized around three key themes: functions and activities, symptom reduction, and quality of life. The results of this study showed that patient expectations regarding a vestibular implant focus on improvement of walking (functions and activities), reduction of oscillopsia (symptom reduction), and being able to live the life before BV developed (quality of life). These expectations seem to be realistic as it aligns with the previous research which showed that a vestibular implant can normalize visual acuity in dynamic conditions and can evoke postural control responses [15–20], thereby encouraging further development of the vestibular implant. Specifically, this study provides a clear guideline on which BV characteristics future vestibular implant research could focus.

The finding that patients expected positive changes regarding their quality of life (e.g., increase of freedom of movement, increase of self-reliance, and increase of overall quality of life) seems to be consistent with results from cochlear implantation research. Potential cochlear implant candidates also expect an increase in self-confidence, to lead a more independent life and daily life activities to become easier and less effortful [32].

Next to this, the connection between themes points out the interrelation of different themes of patient expectations, which is in line with the previous research on patient expectations of treatment outcomes [28]. Patients frequently indicated how improvements of underlying aspects from one theme would affect other themes, with improvement of aspects within the theme “quality of life” often being the ultimate expected goal. This demonstrated that patient expectations were not formulated as delineated or isolated themes, but more in terms of a dynamic interrelated concept, which should be taken into account when measuring these treatment expectations in the future.

It is notable that for some diseases like cancer, treatment expectations focus on survival or extending life expectancy, whereas for other disorders such as chronic low back pain, expectations are more modest [28, 33]. This is in line with the results of this study. BV patients were modest in expressing their expectations and indicated to be satisfied with small improvements. These small improvements, however, proved to be of high value. One possible explanation for the difference between patient expectations for diseases like cancer and patient expectations for disorders like chronic low back pain or BV could be that patient expectations depend on the available treatment and its potential outcome. This could imply that BV patients’ expectations are shaped by the fact that, to date, there is no available causative treatment for BV in clinical practice.

Next to patients’ expectations about vestibular implantation, this study collected patient statements regarding the willingness to accept certain risks related to the surgical procedure. Until now, it remained unclear whether patients with BV are willing to accept a risk factor such as unilateral sensorineural hearing loss in return for restoration of their vestibular function to improve their quality of life [9]. In this study, the majority of the patients indicated deafness (not specified as uni- or bilateral) as an unacceptable risk factor. However, only some patients explicitly indicated single-sided deafness as an unacceptable risk factor, whereas other patients explicitly indicated single-sided deafness as an acceptable risk factor. Previous research has shown that patients with BV have more pronounced negative expectations about possibly having unilateral hearing loss than healthy people and people who are actually suffering from unilateral hearing loss [34]. These latter do not perceive their unilateral hearing loss as a major handicap at all [34, 35]. It is important to acknowledge that the trade-off between improved function and potentials risks related to the surgical procedure (e.g., hearing loss) is difficult. Only when the potential benefits and harms associated with therapeutic choices are known, quality-of-life trade-off can exist. When knowledge on these differences is missing, an informed choice cannot be made [36, 37]. Therefore, proper pre-implantation counseling regarding this subject will be of great importance.

This study provides a base for a future core outcome set to evaluate vestibular implantation in a research setting and in the future clinical practice. One of these outcomes may be “the personal expectation” that can be connected to the key themes of patient expectations discovered in this study. It might be recommended that every suitable vestibular implant candidate state personal expectations prior to implantation and that these expectations are measured on different moments along the implantation and rehabilitation process. This is in accordance with the goal attainment scale, which is a method of scoring the degree to which patient’s individual goals are achieved in the course of intervention [38]. To estimate the extent to which these personal expectations are met, expectations should be formulated according to structured criteria (for instance, SMART criteria) and graded according to a predetermined grading scale [38, 39]. In addition, it might be interesting to monitor how these patients’ expectations develop alongside the evolution of the vestibular implant.

### Limitations

The setting in which these patients were included and interviewed, consisted of a full day of clinical tests. Furthermore, the exclusion criteria comprised being unable to stop medication against anxiety or depression. Due to this setting and these strict exclusion criteria, it cannot be ruled out that this study potentially suffered from selection bias due to the inclusion of a relative “healthy” BV population. As a result, the expectations of patients within the general BV population could be even more distinct compared to this relative healthy BV population.

Next to the main objective of this study, several respondent-driven topics were reported. Since these respondent-driven topics were not inquired in a standardized manner and therefore only assessed in a subgroup of the study population, these findings cannot be generalized for the whole BV population.

Finally, in case a patient indicated hearing loss as an unacceptable risk factor, the interviewer did not always specifically ask for uni- or bilateral hearing loss. As a result, this study potentially underestimates BV patients’ perspectives on accepting risk factors such as unilateral hearing loss in return for restoration of their vestibular function to improve quality of life.

## Conclusion

This study demonstrated that patient expectations regarding a vestibular implant centralize around three key themes: (physical) symptom reduction, functions and activities, and quality of life. These themes closely match the potential functional improvements shown in recent vestibular implantation research. Patients showed modest and realistic expectations for outcomes from treatment, while the reported personal value of the implant was high. Future vestibular implantation research should focus on measuring to which extent patient expectations are met after implantation and rehabilitation (e.g., by means of a goal attainment scale).

## Supplementary Information

Below is the link to the electronic supplementary material.

Supplementary file1 (DOCX 17 KB)

Supplementary file2 (DOC 76 KB)

Supplementary file3 (DOCX 24 KB)

## Data Availability

All included raw data are available upon request.
