# Supplementary material for: Bilateral vestibulopathy patients’ perspectives on vestibular implant treatment: a qualitative study
Source: J Neurol. 2021 Dec 11;269(10):5249–57. doi: 10.1007/s00415-021-10920-z (PMC9467961; doi:10.1007/s00415-021-10920-z)
Supplement: Supplementary file 1 — Supplementary file1 (DOCX 17 KB) [file 415_2021_10920_MOESM1_ESM.docx]

**Online Resource 1 – Journal of Neurology**

**Bilateral Vestibulopathy Patients’ Perspectives on Vestibular Implant Treatment: A Qualitative Study**

Lisa van Stiphout^1^, Florence Lucieer^1^, Nils Guinand^2^ , Angélica Perez Fornos^2^, Maurice van de Berg^1^, Vincent Van Rompaey^3^, Josine Widdershoven^1,3^, Herman Kingma^1^, Manuela Joore^4,5^, Raymond van de Berg^1^

1 Department of Otorhinolaryngology and Head and Neck Surgery, Division of Balance Disorders, Maastricht University Medical Center, School for Mental Health and Neuroscience, Maastricht, Netherlands

2 Service of Otorhinolaryngology Head and Neck Surgery, Department of Clinical Neurosciences, Geneva University Hospitals, Geneva, Switzerland

3 Department of Otorhinolaryngology and Head and Neck Surgery, Antwerp University Hospital, Faculty of Medicine and Health Sciences, University of Antwerp, Antwerp, Belgium.

4 Department of Clinical Epidemiology and Medical Technology Assessment (KEMTA), Maastricht University Medical

5 Care and Public Health Research Institute (CAPHRI), Maastricht University, Maastricht, The Netherlands

**Corresponding author**: Lisa van Stiphout, [lisa.van.stiphout@mumc.nl](mailto:lisa.van.stiphout@mumc.nl)

**Interview Guide**

**Characteristics of the interview**

- Face-to-face and individual interviews
- Location: Maastricht University Medical Center+
- Semi-structured interviews (main question with potential sub-questions, respondent driven topics)
- Planned duration: 60 min
- Digitally audio-recorded
- Language: Dutch

**Aim of the interview**

- To explore and evaluate expectations of patients with bilateral vestibulopathy regarding the vestibular implant.

**Main questions and potential sub-questions**

1. Brief introduction:

- Could you please briefly introduce yourself?
- When were you diagnosed with bilateral vestibulopathy?
- How do you experience living with bilateral vestibulopathy?

1. Inquire about the main topic:

- What are your expectations regarding a vestibular implant?
- Possible follow up questions:
  - What do you expect to gain?
  - Which aspect in life would it affect most? (For example: social, physical, emotional, cognitive, ...)
  - What are your expectations regarding your complaints or symptoms?
  - In which area or situation do you expect a change?
  - In which area or situation do you expect to achieve improvement?
  - What are your expectations regarding your daily functioning?
  - Do you expect changes in your professional or social life activities?
  - Do you expect changes in your professional or social life interactions?

1. Follow up respondent-driven topics (interview dependent).
