# Supplementary material for: Bilateral vestibulopathy patients’ perspectives on vestibular implant treatment: a qualitative study
Source: J Neurol. 2021 Dec 11;269(10):5249–57. doi: 10.1007/s00415-021-10920-z (PMC9467961; doi:10.1007/s00415-021-10920-z)
Supplement: Supplementary file 2 — Supplementary file2 (DOC 76 KB) [file 415_2021_10920_MOESM2_ESM.doc]

**Online Resource 2 – Journal of Neurology**

**Bilateral Vestibulopathy Patients’ Perspectives on Vestibular Implant Treatment: A Qualitative Study**

Lisa van Stiphout^1^, Florence Lucieer^1^, Nils Guinand^2^ , Angélica Perez Fornos^2^, Maurice van de Berg^1^, Vincent Van Rompaey^3^, Josine Widdershoven^1,3^, Herman Kingma^1^, Manuela Joore^4,5^, Raymond van de Berg^1^

1 Department of Otorhinolaryngology and Head and Neck Surgery, Division of Balance Disorders, Maastricht University Medical Center, School for Mental Health and Neuroscience, Maastricht, Netherlands

2 Service of Otorhinolaryngology Head and Neck Surgery, Department of Clinical Neurosciences, Geneva University Hospitals, Geneva, Switzerland

3 Department of Otorhinolaryngology and Head and Neck Surgery, Antwerp University Hospital, Faculty of Medicine and Health Sciences, University of Antwerp, Antwerp, Belgium.

4 Department of Clinical Epidemiology and Medical Technology Assessment (KEMTA), Maastricht University Medical

5 Care and Public Health Research Institute (CAPHRI), Maastricht University, Maastricht, The Netherlands

**Corresponding author**: Lisa van Stiphout, [lisa.van.stiphout@mumc.nl](mailto:lisa.van.stiphout@mumc.nl)

**Consolidated criteria for reporting qualitative studies (COREQ): 32-item checklist**

Developed from:

Tong A, Sainsbury P, Craig J. Consolidated criteria for reporting qualitative research (COREQ): a 32-item checklist for interviews and focus groups. International Journal for Quality in Health Care. 2007. Volume 19, Number 6: pp. 349 – 357

| **No. Item** | **Guide questions/description** | **Reported on Page (section)** |
| --- | --- | --- |
| **Domain 1: Research team and reﬂexivity** | | |
| *Personal Characteristics* | | |
| 1. Interviewer/facilitator | Which author/s conducted the inter view or focus group? | Page 1 and 4 (Title page & Methods section) |
| 2. Credentials | What were the researcher’s credentials? E.g. PhD, MD | Page 1 (Title page) |
| 3. Occupation | What was their occupation at the time of the study? | Page 1 and 4 (Title page & Methods section) |
| 4. Gender | Was the researcher male or female? | Page 1 (Title page) |
| 5. Experience and training | What experience or training did the researcher have? | Page 1 (Title page) |
| *Relationship with participants* | | |
| 6. Relationship established | Was a relationship established prior to study commencement? | Page 4 (Methods section)  . |
| 7. Participant knowledge of the interviewer | What did the participants know about the researcher? e.g. personal goals, reasons for doing the research | Page 4 (Methods section) |
| 8. Interviewer characteristics | What characteristics were reported about the inter viewer/facilitator? e.g. Bias, assumptions, reasons and interests in the research topic | Page 4 (Methods section) |

| **No. Item** | **Guide questions/description** | **Reported on Page (section)** |
| --- | --- | --- |
| **Domain 2: study design** | | |
| *Theoretical framework* | | |
| 9. Methodological orientation and Theory | What methodological orientation was stated to underpin the study? e.g. grounded theory, discourse analysis, ethnography, phenomenology, content analysis | Page 1 and 4 (Title page & Methods section) |
| *Participant selection* | | |
| 10. Sampling | How were participants selected? e.g. purposive, convenience, consecutive, snowball | Page 3 (Methods section) |
| 11. Method of approach | How were participants approached? e.g. face-to-face, telephone, mail, email | Page 3 (Methods section) |
| 12. Sample size | How many participants were in the study? | Page 4 (Results section) |
| 13. Non-participation | How many people refused to participate or dropped out? Reasons? | Page 3 (Methods section) |
| *Setting* | | |
| 14. Setting of data collection | Where was the data collected? e.g. home, clinic, workplace | Page 3 and 4 (Methods section) |
| 15. Presence of non-participants | Was anyone else present besides the participants and researchers? | Page 3 and 4 (Methods section) |
| 16. Description of sample | What are the important characteristics of the sample? e.g. demographic data, date | Page 4 (Results section) |
| *Data collection* | | |
| 17. Interview guide | Were questions, prompts, guides provided by the authors? Was it pilot tested? | Page 3 and 4 (Methods section & Supplementary Material) |
| 18. Repeat interviews | Were repeat inter views carried out? If yes, how many? | No (inferred from page 3 and 4, Methods section). |
| 19. Audio/visual recording | Did the research use audio or visual recording to collect the data? | Page 4 (Methods section) |
| 20. Field notes | Were ﬁeld notes made during and/or after the inter view or focus group? | Page 4 (Methods section) |
| 21. Duration | What was the duration of the inter views or focus group? | Page 4 (Methods section) |
| 22. Data saturation | Was data saturation discussed? | Page 4 (Methods section) |
| 23. Transcripts returned | Were transcripts returned to participants for comment and/or correction? | No |

| **No. Item** | **Guide questions/description** | **Reported on Page (section)** |
| --- | --- | --- |
| **Domain 3: analysis and ﬁndings** | | |
| *Data analysis* | | |
| 24. Number of data coders | How many data coders coded the data? | Page 4 (Methods section) |
| 25. Description of the coding tree | Did authors provide a description of the coding tree? | Page 4 (Methods section) |
| 26. Derivation of themes | Were themes identiﬁed in advance or derived from the data? | Page 4 (Methods section) |
| 27. Software | What software, if applicable, was used to manage the data? | Page 4 (Methods section) |
| 28. Participant checking | Did participants provide feedback on the ﬁndings? | No |
| *Reporting* | | |
| 29. Quotations presented | Were participant quotations presented to illustrate the themes/ﬁndings? Was each quotation identiﬁed? e.g. participant number | Page 4 - 7 (Results section) |
| 30. Data and ﬁndings consistent | Was there consistency between the data presented and the ﬁndings? | Yes, there was.  Page 4 - 8 (Results & Discussion section) |
| 31. Clarity of major themes | Were major themes clearly presented in the ﬁndings? | Yes. they were.  Page 4 - 8 (Results & Discussion section) |
| 32. Clarity of minor themes | Is there a description of diverse cases or discussion of minor themes? | Page 4 - 8 (Results & Discussion section) |
