# Supplementary material for: Bilateral vestibulopathy patients’ perspectives on vestibular implant treatment: a qualitative study
Source: J Neurol. 2021 Dec 11;269(10):5249–57. doi: 10.1007/s00415-021-10920-z (PMC9467961; doi:10.1007/s00415-021-10920-z)
Supplement: Supplementary file 3 — Supplementary file3 (DOCX 24 KB) [file 415_2021_10920_MOESM3_ESM.docx]

**Online Resource 3 – Journal of Neurology**

**Bilateral Vestibulopathy Patients’ Perspectives on Vestibular Implant Treatment: A Qualitative Study**

Lisa van Stiphout^1^, Florence Lucieer^1^, Nils Guinand^2^ , Angélica Perez Fornos^2^, Maurice van de Berg^1^, Vincent Van Rompaey^3^, Josine Widdershoven^1,3^, Herman Kingma^1^, Manuela Joore^4,5^, Raymond van de Berg^1^

1 Department of Otorhinolaryngology and Head and Neck Surgery, Division of Balance Disorders, Maastricht University Medical Center, School for Mental Health and Neuroscience, Maastricht, Netherlands

2 Service of Otorhinolaryngology Head and Neck Surgery, Department of Clinical Neurosciences, Geneva University Hospitals, Geneva, Switzerland

3 Department of Otorhinolaryngology and Head and Neck Surgery, Antwerp University Hospital, Faculty of Medicine and Health Sciences, University of Antwerp, Antwerp, Belgium.

4 Department of Clinical Epidemiology and Medical Technology Assessment (KEMTA), Maastricht University Medical

5 Care and Public Health Research Institute (CAPHRI), Maastricht University, Maastricht, The Netherlands

**Corresponding author**: Lisa van Stiphout, [lisa.van.stiphout@mumc.nl](mailto:lisa.van.stiphout@mumc.nl)

**Table 1** Specification of reported expectations of patients with bilateral vestibulopathy regarding the vestibular implant, divided into three key domains: functions and activities, symptom reduction and quality of life. The domains are divided into categories and subsequently into sub-categories. For each domain, category or sub-category, it is indicated how often it was mentioned by the patients (frequency, n=48). It was possible for patients to mention more than one expectation

| **Main domain** | **Category** | **Sub-category** | **Frequency** |
| --- | --- | --- | --- |
| Functions & Activities |  |  | 77 |
|  | Walking |  | 28 |
|  |  | In a straight line | 8 |
|  |  | Without worrying | 6 |
|  |  | Unspecified | 6 |
|  |  | On uneven ground | 3 |
|  |  | In the dark | 3 |
|  |  | Without walking aid | 1 |
|  |  | Without correction | 1 |
|  | Transportation |  | 18 |
|  |  | Driving a car | 11 |
|  |  | Going on holiday | 4 |
|  |  | Unspecified | 2 |
|  |  | Using public transportation | 1 |
|  | Cycling |  | 13 |
|  |  | Unspecified | 12 |
|  |  | In the dark | 1 |
|  | Doing sports |  | 13 |
|  |  | Hiking | 5 |
|  |  | Tennis | 3 |
|  |  | Dancing | 2 |
|  |  | Playing soccer | 1 |
|  |  | Gymnastics | 1 |
|  |  | Skating | 1 |
|  | Working |  | 4 |
|  | Making Music |  | 1 |
| Symptom Reduction |  |  | 24 |
|  | Oscillopsia |  | 13 |
|  |  | During locomotion | 5 |
|  |  | Blurred vision | 4 |
|  |  | Shaky feeling of the head | 3 |
|  |  | Unspecified | 1 |
|  | Balance |  | 5 |
|  | Restlessness |  | 3 |
|  | Tiredness |  | 2 |
|  | Nausea |  | 1 |
| Quality of Life |  |  | 24 |
|  | Being able to live the life from before the development of BV |  | 8 |
|  | Freedom of movement |  | 7 |
|  | Being independent |  | 5 |
|  | Doing activities without thinking |  | 2 |
|  | Unspecified |  | 2 |

**Table 2** Specification of the desired level of overall improvement after receiving a vestibular implant of patients with bilateral vestibulopathy (respondent driven topic). For each category, it is indicated how often it was mentioned by the patients (frequency, n=16)

| **Category** | **Frequency** |
| --- | --- |
| All minimal improvement is desired | 5 |
| Enough improvement to be able to manage daily life | 1 |
| Improvement of 1 symptom | 2 |
| 50% improvement | 3 |
| 60-70% improvement | 2 |
| 100% improvement | 3 |

**Table 3** Number of patients with bilateral vestibulopathy (frequency, n=19) who would or would not consider a vestibular implant at the moment of the conducted interview (respondent driven topic)

| **Category** | **Frequency** |
| --- | --- |
| Would consider VI treatment | 15 |
| Would not consider VI treatment | 4 |

**Table 4** Specification of reported estimated (personal) value of the vestibular implant of patients with bilateral vestibulopathy (respondent driven topic). For each category, it is indicated how often it was mentioned by the patients (frequency, n=41)

| **Category** | **Frequency** |
| --- | --- |
| Priceless | 3 |
| Everything | 5 |
| A fortune | 1 |
| Taking out a new loan/mortgage | 3 |
| Selling shares or land | 2 |
| 1.000.000 | 3 |
| 100.000-500.000 | 4 |
| 10.000-100.000 | 15 |
| >500-3000 | 2 |
| Insurance | 2 |
| Not worth everything | 1 |

**Table 5** Specification of reported expected or acceptable number of post-operative care consultations of patients with bilateral vestibulopathy after vestibular implant treatment (respondent driven topic). For each category, it is indicated how often it was mentioned by the patients (frequency, n=18)

| **Category** | **Frequency** |
| --- | --- |
| Whatever it takes | 8 |
| Weekly | 5 |
| Two-weekly | 1 |
| Monthly | 2 |
| Half-yearly | 2 |

**Table 6** Specification of reported acceptable and unacceptable surgical risks of patients with bilateral vestibulopathy regarding the vestibular implant treatment (respondent driven topic). The two categories are divided into subcategories. For each subcategory, it is indicated how often it was mentioned by the patients (frequency, n=27)

| **Category** | **Sub-category** | **Sub-category II** | **Frequency** |
| --- | --- | --- | --- |
| Acceptable risks |  |  | 5 |
|  | Unilateral hearing loss |  | 4 |
|  | Infections |  | 1 |
| Unacceptable risks |  |  | 22 |
|  | Hearing loss |  | 16 |
|  |  | Unspecified | 12 |
|  |  | Unilateral hearing loss | 2 |
|  |  | Bilateral hearing loss | 2 |
|  | Other organ damage |  | 6 |
|  |  | Infections | 2 |
|  |  | Unspecified | 1 |
|  |  | Amnesia | 1 |
|  |  | Blindness | 1 |
|  |  | Paralysis | 1 |
